# P-2246. Diagnostic Performance of a Rapid Host-Protein Test (MeMed BV) In Detecting Bacteremia in Children

**DOI:** 10.1093/ofid/ofae631.2399

**Published:** 2025-01-29

**Authors:** Lior Kellerman, Eran Eden, Tanya Gottlieb, Roy Navon, Michal Stein, Isaac Srugo, Susanna Esposito, Cihan Papan, Tobias Tenenbaum, Louis J Bont, Adi Klein, Richard Bachur

**Affiliations:** MeMed, Haifa, Israel, Haifa, Hefa, Israel; MeMed Diagnostics, Tirat Carmel, HaZafon, Israel; MeMed Diagnostics, Tirat Carmel, HaZafon, Israel; MeMed Diagnostics, Tirat Carmel, HaZafon, Israel; Sheba Medical Center, Tel HaShomer, HaMerkaz, Israel; Bnai-Zion Medical Center, Haifa, HaZafon, Israel; Pediatric Clinic, Pietro Barilla Children’s Hospital, Parma, Emilia-Romagna, Italy; University Hospital Bonn, Bonn, Nordrhein-Westfalen, Germany; Sana Klinikum Lichtenberg Berlin, Berlin, Berlin, Germany; University Medical Centre Utrecht, Utrecht, Utrecht, Netherlands; Hillel Yaffe Medical Center, Hedera, HaZafon, Israel; Boston Children's Hospital, Boston, Massachusetts

## Abstract

**Background:**

The gold standard for detecting infections associated with bacteremia is blood cultures. Blood culture yield is low, contamination is frequent and results take 12-48 hours.

MeMed BV® is a rapid, FDA-cleared, host-protein test to differentiate bacterial and viral infection, with sensitivity and specificity >90%, and negative predictive value >98%. Here, we assessed its performance in detecting bacteremia in children as early detection of invasive infections is paramount.

**Figure:**
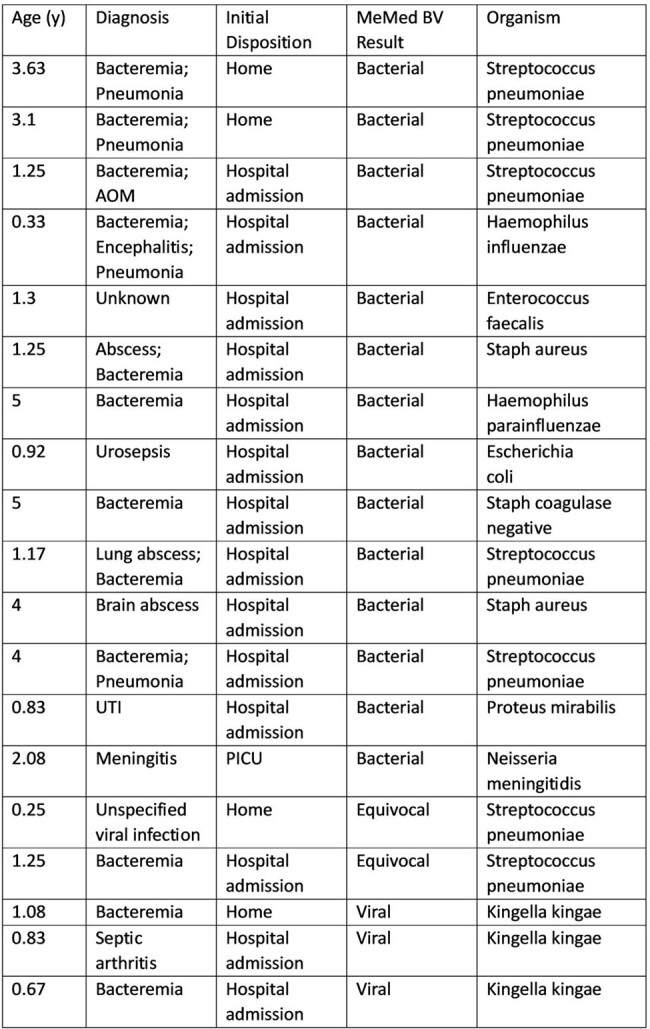

**Methods:**

Multi-cohort analysis of 7 prospective diagnostic accuracy studies of febrile children with suspected infection at the Emergency Department and inpatients. Eligibility required inclusion in the original study, age 3 months to 5 years and blood culture ordered.

Reference standard infection etiology was adjudicated by 3 experts based on review of comprehensive patient data including clinical outcomes. Experts were blinded to MeMed BV results. Cases with bacterial reference standard and positive blood cultures adjudicated as having non-contaminant growth were defined as bacteremia.

**Results:**

1987 patients were studied. The median age was 1.3 years (interquartile range 0.8-2.4); 54.6% were male and 46.7% were hospitalized. The most common ED discharge diagnoses were viral infection (33.6%), upper respiratory tract infection (20.0%), lower respiratory tract infection (16.1%). The prevalence of bacterial infection was 15.4%. MeMed BV diagnostic performance was evaluated against the reference standard and attained sensitivity of 89.9% (95%CI 85.6-93.0), specificity of 94.3% (95%CI 93.0-95.4), and NPV of 98.1% (95%CI 97.3-98.7) for identifying bacterial infection.

19 (1%) patients had bacteremia; among these, MeMed BV identified 14 with bacterial scores, 2 with equivocal scores, and 3 with viral scores, the latter positive for *Kingella kingae* (see Table for more details).

**Conclusion:**

MeMed BV can aid in early detection of bacteremia in children.

**Disclosures:**

Lior Kellerman, M.D, MeMed: Employee|MeMed: Stocks/Bonds (Private Company) Eran Eden, PhD, MeMed: Employee|MeMed: Stocks/Bonds (Private Company) Tanya Gottlieb, PhD, MeMed: Employee|MeMed: Stocks/Bonds (Private Company) Roy Navon, MSc, MeMed: Employee|MeMed: Stocks/Bonds (Private Company) Richard Bachur, MD, MeMed: Advisor/Consultant|UpToDate - WoltersKluwer: Advisor/Consultant|UpToDate - WoltersKluwer: Royalties - Editor

